# The Efficacy and Safety of Mainstream Medications for Patients With cDMARD-Naïve Rheumatoid Arthritis: A Network Meta-Analysis

**DOI:** 10.3389/fphar.2018.00138

**Published:** 2018-03-21

**Authors:** Weiyan Cai, Youyi Gu, Huanqin Cui, Yinyin Cao, Xiaoliang Wang, Yi Yao, Mingyu Wang

**Affiliations:** ^1^Department of Pediatrics, Yantai Yuhuangding Hospital Affiliated to Qingdao Medical College of Qingdao University, Yantai, China; ^2^Department of Integrated Chinese and Western Medicine, Yantai Yuhuangding Hospital Affiliated to Qingdao Medical College of Qingdao University, Yantai, China; ^3^Department of Rheumatology and Immunology, Yantai Yuhuangding Hospital Affiliated to Qingdao Medical College of Qingdao University, Yantai, China

**Keywords:** rheumatoid arthritis, DMARDs, safety, efficacy, network meta-analysis

## Abstract

**Background:** The mainstream medications for rheumatoid arthritis (RA) include conventional disease-modifying antirheumatic drugs (cDMARDs), which mostly are methotrexate (MTX), and biologic agents such as adalimumab (ADA), certolizumab (CZP), etanercept (ETN), golimumab (GOL), infliximab (IFX), and tocilizumab (TCZ). This network meta-analysis was aimed at evaluating the efficacy and safety of the medications above and interventions combining cDMARDs and biologic agents for patients with RA.

**Methods:** PubMed, EMBASE, Cochrane Library, and ClinicalTrials.gov were searched systematically for eligible randomized controlled trials (RCTs). Outcomes concerning efficacy and safety were evaluated utilizing odds ratios (ORs) and 95% credible intervals (*CrI*). The outcomes of efficacy would be evaluated through remission and American College of Rheumatology (ACR) scores. The surface under the cumulative ranking curve (SUCRA) was calculated to rank each treatment on each index.

**Results:** A total of 20 RCTs with 9,047 patients were included, and the efficacy and safety of the concerning interventions for RA were evaluated. Compared with cDMARDs alone, TCZ+MTX, ETN+MTX, IFX+MTX, TCZ, and ADA+MTX showed significant statistical advantage on ACR20, ACR50, and ACR70. Apart from that, as for remission, TCZ+MTX, IFX+MTX, TCZ, and CZP+MTX performed better compared to cDMARDs alone. The SUCRA ranking also indicated that TCZ+MTX was the intervention with best ranking in the entire four efficacy indexes followed by ETX+MTX and IFX+MTX. However, there was no obvious difference among these medications compared with cDMARDs when it comes to safety, which need more specific studies on that.

**Conclusion:** TCZ+MTX was potentially the most recommended combination of medications for RA due to its good performance in all outcomes of efficacy. ETX+MTX and IFX+MTX, which also performed well, could be introduced as alternative treatments. However, considering the adverse events, the treatments concerning should be introduced with caution.

## Introduction

Rheumatoid arthritis (RA) is a chronic inflammatory autoimmune disease characterized by its irreversible, alternating episodes and impaired joint function (Popescu et al., 1985). Patients with RA often suffered from the arthralgia caused by the synovial lining joints swelling which can result in disability and reduction of life quality (Donahue et al., 2012). Generally, patients with RA often have a shorter life expectancy compared with normal people. Thus, the primary treating target of RA patients is to maximize the quality of life associated with health through preventing structural damage, controlling the symptom of inflammation, normalizing functional, and social participation (Smolen et al., 2014; Buckley et al., 2015). Until now, there are an estimated 1.12% of adult people affected with RA in developed countries (Li et al., 2012; Stevenson et al., 2016) which leads us to find optional treatments for patients with this disease.

Recently, the potent pro-inflammatory cytokine named tumor necrosis factor-α (TNF-α) has been considered playing an important role in immune responses and inflammationincluding those involved in RA (Brennan et al., 1992), Which indicated that TNF antagonists could be an effective method for RA treatments (Lee and Bae, 2016). However, based on the American College of Rheumatology (ACR) recommendations for the treatment of RA, it should begin with the use of conventional (non-biologic) disease-modifying antirheumatic drugs (cDMARDs), mostly are methotrexate (MTX) (Singh et al., 2012). If patients were tolerant of cDMARDs or showed inadequate responses (IR), biologic agents were often applied with cDMARDs as combined therapies. On the other hand, because of cDMARDs' side effects including hepatotoxicity, primary gastrointestinal symptoms and respiratory symptoms, around one-third RA patients are treated with monotherapy of biologic agents (Listing et al., 2006; Heiberg et al., 2008; Soliman et al., 2011). Up to now, a total of five kind of biologic agents have been approved to treat patients with RA: (Popescu et al., 1985) TNF antagonists, known as anti-TNF agents (aTNF) including infliximab (IFX), certolizumab (CZP), adalimumab (ADA), golimumab (GOL), and etanercept (ETN); (Donahue et al., 2012) monoclonal antibody which could suppress B cells such as rituximab; (Buckley et al., 2015) monoclonal antibody which could suppress interleukin-6 (IL-6) receptor such as tocilizumab (TCZ); (Smolen et al., 2014) selective T-cell costimulatory modulator such as abatacept; (Stevenson et al., 2016) interleukin-1 (IL-1) receptor antagonists such as anakinra (Buckley et al., 2015).

However, no randomized controlled trial (RCT) has been conducted to evaluate all optional biologic treatments simultaneously. Clinicians now were facing increasing challenge about choosing optimal drug due to the amount of alternative biologic treatments and other DMARDs. Thus, network meta-analysis (NMA) has been applied, which could combine all the available RCTs and evaluate the potential biologic drugs through not only direct but also indirect comparison. In recent years, several NMAs of biologic treatments for patients with RA have been published (Buckley et al., 2015; Lee and Bae, 2016; Migliore et al., 2016; Stevenson et al., 2016; Choi et al., 2017). Nevertheless, those studies only focused on combined treatments such as biologic therapies with MTX. Particularly, none of the existing NMA contained all optional biologic agents. Besides, none of the existing NMA distinguished between cDMARD-naive and cDMARD-experienced. According to Egsmose et al., Tsaknoas et al., and Quinn et al., there is a period named “window of opportunity” and the underlying process of inflammatory in RA was more susceptible to biologic drugs than later time-points (Egsmose et al., 1995; Tsakonas et al., 2000; Quinn et al., 2001). In the early period of RA, the mechanical aspects and pathogenic of autoimmune prompted inflammation was not fully consistent with the current evidence of RA (Mullan and Bresnihan, 2003). Correspondingly, the RCTs on patients with RA who are cDMARD-naive should be picked out from the pool.

In this study, a comprehensive NMA was conducted to evaluate the relative efficacy and safety of 11 potential therapeutic approaches of early interventions for patients with RA. The outcomes of efficacy would be evaluated through remission and ACR scores. ACR scores that measuring changes in symptoms of RA and different degrees of improvement are referred to ACR20, ACR50, and ACR70. For example, ACR20 measures a 20% improvement on a scale of 28 intervals. As for safety, the outcomes would consist of the incidence of adverse effects (AEs) and serious adverse effects (SAEs). The purpose of this current research is to supplement the existing evidence network and select the optimal treatments for patients with RA.

## Methods

### Selection strategy

We did a comprehensive research to find all relevant RCTs through Embase, PubMed, Cochrane Library and ClinicalTrials.gov with the following keywords: “rheumatoid arthritis,” “methotrexate,” “infliximab,” “etanercept,” “adalimumab,” “golimuma,” “tocilizumab,” and “randomized control trial,” etc. There is no limitation on the time of publication. All the searching process was limited to clinical trials, and no age or language restrictions were applied to literature search. Process of screening was carried out through reading titles and abstracts of eligible articles. After that, full texts of remaining articles were further read to remove articles with incomplete or irrelevant information.

### Inclusion and exclusion criteria

All the included articles must satisfy the following criteria: (i) studies should be randomized and all involved RA patients were adults who had not treat with cDMARDs before trials; (ii) trials must include at least two of the concerning treatments for RA; (iii) trials should contain at least one of the primary outcomes of interest (as shown below). Moreover, expert opinions, editorials, letters, case reviews, reports and duplications would be excluded after title and abstract screening.

### Data extraction and quality assessment

All the relevant data would be extracted from the eligible studies by two independent reviewers using a standard data collection form. Any discrepancies between reviewers would be resolved by discussing with a third independent researcher. In this study, the following information would be collected: (i) baseline information including first author, publication date, sample size, blinding method, type of intervention, following time, disease durations, gender, and age; (ii) efficacy outcomes including ACR20, ACR50, ACR70, and remission; (iii) safety outcomes including AEs and SAEs.

### Statistical analysis

On the foundation of a rigorous assessment of the accuracy and authenticity of the collected data, we introduced a Bayesian framework utilizing Software R 3.2.3 and STATA 13.0 for statistical processing. One of the most significant properties of NMA is to combine all the available comparisons including the indirect evidence simultaneously. In this NMA research, the forest plots showed the results of each outcome. For binary variables (ACR20, ACR50, ACR70, remission, AEs, SAEs), odds ratios (ORs) with their 95% credible intervals (CrI) were applied for the comparison. Furthermore, relative ranking probability of each therapeutic method was calculated through surface under cumulative ranking curve (SUCRA), which is also an advantage of the Bayesian framework. Typically, a more satisfying treatment assessed under a certain outcome was indicated by a higher SUCRA value. As for the consistency analysis, the consistency between direct and indirect evidence of each outcome were conducted through node-splitting analysis and heat plots. Besides, random effects model would be implemented if significant inconsistency was found (*P*-value < 0.05).

## Results

### Study selection and characteristics of included studies

**Figure 1:**
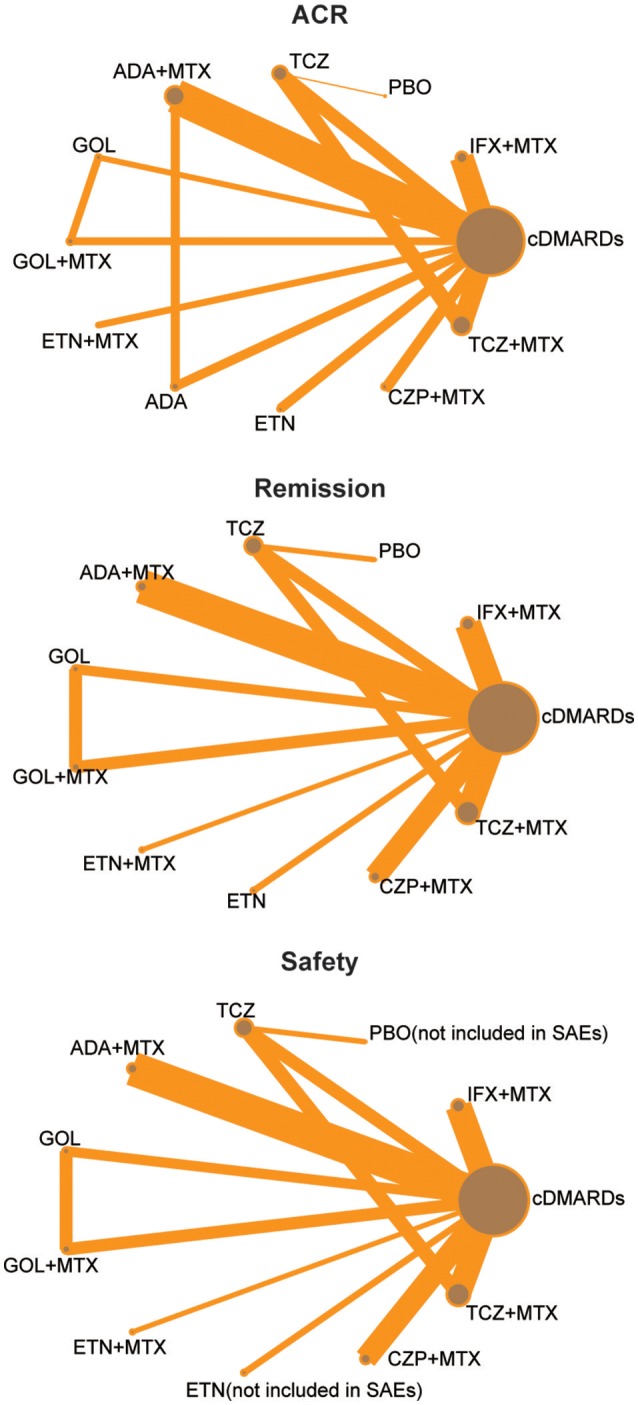
Full network of comparisons of efficacy and safety outcomes (The width of the lines is proportional to the number of trials comparing each pair of treatments; the area of circles represents the cumulative number of patients for each intervention).

We identified 2,527 published articles according to the searching strategy which has been mentioned before. Then 864 articles were removed for duplicated, and after scanning title and abstract 1,427 articles were also removed for lack of relevance. 216 studies were eventually excluded. At the end, a total of 20 studies published from 2000 to 2016 met our selection criteria and has been involved in this NMA (Bathon et al., 2000; Nishimoto et al., 2004; St Clair et al., 2004; Quinn et al., 2005; Breedveld et al., 2006; Durez et al., 2007; Bejarano et al., 2008; Emery et al., 2008a,b, 2009, 2016; Goekoop-Ruiterman et al., 2008; Soubrier et al., 2009; van Vollenhoven et al., 2009; Burmester et al., 2013; Detert et al., 2013; Kavanaugh et al., 2013; Hørslev-Petersen et al., 2014; Atsumi et al., 2015; Bijlsma et al., 2016), among which 12 were two-arm trials, 6 were three-arm trials and 2 were four-arm trials. The baseline characteristics of included studies were presented in Table 1. Overall, 9,047 patients with RA were contained and the average age of them was 52.4 ± 10 years. The network structures of ACR scores, remission and safety were shown in Figure 1 and in the diagram, each circle represents an individual treatment and the thickness of lines represents the number of trials.

**Table 1:** Patient characteristics in the studies included in the analysis.

**Study, first author, year**	**Blinding**	**Follow up (weeks)**	**Sizes**	**Outcomes**	**Intervention**	**Cases**	**Female**	**Disease durations (year)**	**Age (year)**
Swefot (TBD), van Vollenhoven et al., 2009	Open-label	52	258	①③④	cDMARDs	130	101	0.525 (0.3)	53.9 (13.9)
					IFX+MTX	128	97	0.517 (0.29)	51.1 (13.3)
Nishimoto, 2004, Nishimoto et al., 2004	Double-blind	12	162	①③	PBO	53	39	8.4	53
					TCZ	54	40	7.3	53.5
					TCZ	55	46	8.3	56
GUEPARD, Soubrier et al., 2009	Unblinded	52	65	①②	cDMARDs	32	26	4.4	49.3 (15.2)
					ADA+MTX	33	26	4.4	46.3 (16.3)
ASPIRE, St Clair et al., 2004	Not specified	46	1,004	①②③④	cDMARDs	282	212	0.9 (0.7)	50 (13)
					IFX+MTX	359	255	0.8 (0.7)	51 (12)
					IFX+MTX	363	247	0.9 (0.8)	50 (130)
OPTIMA, Kavanaugh et al., 2013	Double-blind	26	1,032	①②③④	ADA+MTX	515	380	0.33 (0.3)	50.7 (14.5)
					cDMARDs	517	382	0.375 (0.6)	50.4 (13.6)
BeST, Goekoop-Ruiterman et al., 2008	Double-blind	26	254	①③④	cDMARDs	126	86	0.44	54 (13)
					IFX+MTX	128	85	0.62	54 (14)
GO-BEFORE, Emery et al., 2009	Double-blind	24	637	①②③④	cDMARDs	160	134	2.9 (4.80)	48.6 (12.91)
					GOL	159	134	4.1 (5.60)	48.2 (12.85)
					GOL+MTX	159	135	3.5 (5.65)	50.9 (11.32)
					GOL+MTX	159	125	3.6 (6.09)	50.2 (11.87)
COMET, Emery et al., 2008a	Double-blind	104	528	①②③④	cDMARDs	263	191	0.78 (0.03)	52.3 (0.8)
					ETN+MTX	265	196	0.73 (0.03)	50.5 (0.9)
Durez2007, Durez et al., 2007	Double-blind	52	29	①	cDMARDs	14	10	0.45 (0.29)	53.8 (15.2)
					IFX+MTX	15	5	0.36 (0.31)	50.0 (9.9)
PREMIER, Breedveld et al., 2006	Double-blind	104	799	①②	ADA+MTX	268	193	0.7 (0.8)	51.9 (14.0)
					ADA	274	212	0.7 (0.8)	52.1 (13.5)
					cDMARDs	257	190	0.8 (0.9)	52.0 (13.1)
Bathon, 2000, Bathon et al., 2000	Double-blind	52	632	①③	cDMARDs	217	163	1 (0.92)	49 (13)
					ETN	208	156	0.92 (0.83)	50 (13)
					ETN	207	153	1 (0.92)	51 (13)
Bejarano, 2008, Bejarano et al., 2008	Double-blind	56	128	①②③④	cDMARDs	73	39	0.66 (0.45)	47 (9)
					ADA+MTX	75	44	0.79 (0.5)	47 (9)
HITHARD, Detert et al., 2013	Double-blind	48	172	①②④	ADA+MTX	87	61	0.15 (0.17)	47.2 (12.12)
					cDMARDs	85	57	0.13 (0.14)	52.5 (14.34)
Quinn, 2005, Quinn et al., 2005	Double-blind	52	20	①②	IFX+MTX	10	N/A	0.62 (0.38)	51.3 (9.5)
					cDMARDs	10	N/A	0.5 (0.31)	53.1 (13.7)
OPERA, Hørslev-Petersen et al., 2014	Double-blind	52	180	①④	ADA+MTX	89	56	88 days	56.2
					cDMARDs	91	63	83 days	54.2
C-EARLY, Emery et al., 2016	Double-blind	52	868	①②③④	cDMARDs	213	170	0.24 (0.24)	51.2 (13.0)
					CZP+MTX	655	497	0.24 (0.38)	50.4 (13.6)
U-Act-Early, Bijlsma et al., 2016	Double-blind	24	317	①③④	TCZ+MTX	106	65	24.5 days	53
					TCZ	103	78	25.5 days	55
					cDMARDs	108	69	27.0 days	53.5
Function, Burmester et al., 2013	Double-blind	52	1,157	①②③④	cDMARDs	287	229	0.4 (0.48)	49.6 (13.1)
					TCZ+MTX	288	228	0.4 (0.49)	51.2 (13.84)
					TCZ+MTX	290	228	0.5 (0.53)	49.5 (13.70)
					TCZ	292	219	0.5 (0.48)	49.9 (13.22)
C-OPERA, Atsumi et al., 2015	Double-blind	24	316	②③④	cDMARDs	157	127	N/A	49 (10.3)
					CZP+MTX	159	129	N/A	49.4 (10.6)
RADIATE, Emery et al., 2008b	Double-blind	24	489	①②③④	TCZ+MTX	170	84	12.6 (9.3)	53.9 (12.7)
					TCZ+MTX	161	81	11.0 (8.5)	50.9 (12.5)
					cDMARDs	158	79	11.4 (9.2)	53.4 (13.3)

*① ACR*.

*② Remission*.

*③ adverse effects (AEs)*.

*④ serious adverse effects*.

*cDMARDs: conventional disease-modifying antirheumatic drugs; MTX: methotrexate; ADA: adalimumab; CZP: certolizumab; ETN: etanercept; GOL: golimumab; IFX: infliximab; TCZ: PBO: tocilizumab; placebo*.

### Network meta-analysis

**Figure 2:**
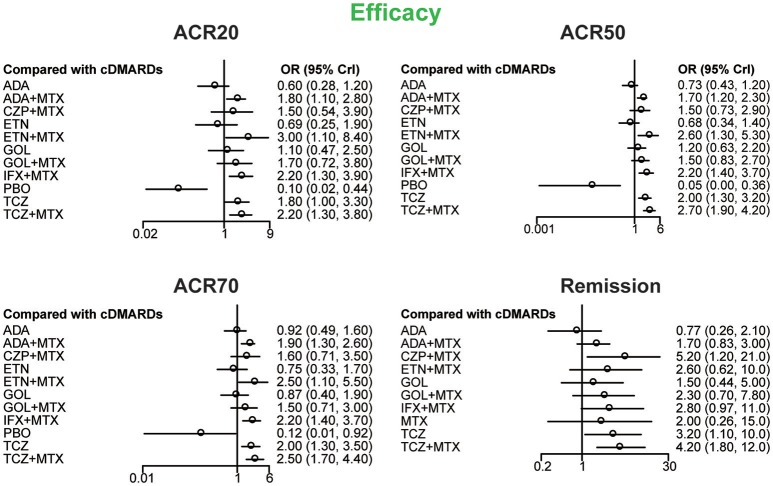
The Odds ratio estimate with 95% credible intervals of efficacy endpoints compared to DMARDs.

ACR20, ACR50, ACR70, and remission were used to estimate the effectiveness of each therapeutic method and the results were shown in Figure 2. According to the results of ACR scores, TCZ+MTX, IFX+MTX, and ETN+MTX showed statistical difference compared with cDMARDs alone on ACR20, ACR50, and ACR70, which indicated the superior efficacy of these drug combinations. Apart from that, as for remission, CZP+MTX (OR = 5.20, 95% CrI: 1.20–21.0), TCZ (OR = 3.20, 95% CrI: 1.10–10.0), TCZ+MTX (OR = 4.20, 95% CrI: 1.80–12.0) were statistically significant compared to cDMARDs. Tables 2, 3 showed the results of safety outcomes on AEs and SAEs, and according to that, there is no significant difference of the concerning treatments compared with cDMARDs and PBO.

**Table 2:** The odds ratio estimate with 95% credible intervals of AEs for each pair-wise comparison.

**ADA+MTX**	0.84 (0.38, 1.8)	1.20 (0.41, 3.56)	0.40 (0.10, 1.51)	0.79 (0.20, 3.16)	0.66 (0.21, 2.03)	1.17 (0.37, 3.63)	1.09 (0.39, 2.86)	0.97 (0.19, 4.26)	0.91 (0.29, 2.39)	1.40 (0.52, 3.74)
1.19 (0.55, 2.61)	**cDMARDs**	1.42 (0.68, 3.06)	0.47 (0.16, 1.42)	0.95 (0.30, 2.97)	0.78 (0.34, 1.84)	1.40 (0.60, 3.19)	1.30 (0.69, 2.34)	1.15 (0.28, 4.10)	1.08 (0.49, 2.05)	1.67 (0.90, 3.06)
0.84 (0.28, 2.46)	0.70 (0.33, 1.48)	**CZP+MTX**	0.33 (0.09, 1.26)	0.66 (0.17, 2.59)	0.55 (0.18, 1.70)	0.98 (0.31, 2.94)	0.91 (0.34, 2.34)	0.81 (0.16, 3.53)	0.76 (0.25, 1.99)	1.16 (0.44, 3.06)
2.51 (0.66, 9.78)	2.12 (0.70, 6.30)	3.00 (0.79, 11.7)	**ETN**	2.01 (0.41, 9.78)	1.65 (0.42, 6.49)	2.97 (0.75, 11.82)	2.75 (0.76, 9.49)	2.44 (0.40, 13.07)	2.29 (0.58, 8.00)	3.53 (1.00, 12.43)
1.26 (0.32, 5.05)	1.05 (0.34, 3.32)	1.51 (0.39, 5.93)	0.50 (0.1, 2.41)	**ETN+MTX**	0.83 (0.20, 3.39)	1.48 (0.36, 6.05)	1.38 (0.37, 4.90)	1.22 (0.20, 6.62)	1.14 (0.28, 4.10)	1.75 (0.49, 6.49)
1.52 (0.49, 4.85)	1.28 (0.54, 2.97)	1.82 (0.59, 5.70)	0.61 (0.15, 2.39)	1.21 (0.30, 5.00)	**GOL**	1.79 (0.78, 4.10)	1.67 (0.57, 4.57)	1.48 (0.28, 6.69)	1.39 (0.43, 3.82)	2.12 (0.76, 5.99)
0.85 (0.28, 2.72)	0.71 (0.31, 1.67)	1.02 (0.34, 3.19)	0.34 (0.08, 1.34)	0.68 (0.17, 2.80)	0.56 (0.24, 1.28)	**GOL+MTX**	0.93 (0.32, 2.59)	0.83 (0.16, 3.78)	0.78 (0.24, 2.16)	1.19 (0.43, 3.39)
0.91 (0.35, 2.56)	0.77 (0.43, 1.45)	1.09 (0.43, 2.97)	0.36 (0.11, 1.31)	0.73 (0.20, 2.72)	0.60 (0.22, 1.77)	1.07 (0.39, 3.10)	**IFX+MTX**	0.89 (0.19, 3.67)	0.84 (0.31, 2.01)	1.27 (0.55, 3.13)
1.03 (0.23, 5.26)	0.87 (0.24, 3.6)	1.23 (0.28, 6.30)	0.41 (0.08, 2.51)	0.82 (0.15, 5.10)	0.68 (0.15, 3.53)	1.21 (0.26, 6.36)	1.13 (0.27, 5.21)	**PBO**	0.93 (0.30, 2.92)	1.43 (0.41, 5.99)
1.09 (0.42, 3.46)	0.92 (0.49, 2.03)	1.31 (0.50, 4.06)	0.44 (0.12, 1.73)	0.88 (0.24, 3.56)	0.72 (0.26, 2.34)	1.28 (0.46, 4.18)	1.20 (0.50, 3.22)	1.07 (0.34, 3.32)	**TCZ**	1.52 (0.82, 3.39)
0.71 (0.27, 1.93)	0.60 (0.33, 1.11)	0.86 (0.33, 2.27)	0.28 (0.08, 1.00)	0.57 (0.15, 2.05)	0.47 (0.17, 1.31)	0.84 (0.3, 2.34)	0.79 (0.32, 1.8)	0.70 (0.17, 2.46)	0.66 (0.30, 1.22)	**TCZ+MTX**

*cDMARDs, conventional disease-modifying antirheumatic drugs; MTX, methotrexate; ADA, adalimumab; CZP, certolizumab; ETN, etanercept; GOL, golimumab; IFX, infliximab; TCZ, tocilizumab; PBO, placebo*.

**Table 3:** The odds ratio estimate with 95% credible intervals of SAEs for each pair-wise comparison.

**ADA+MTX**	1.00 (0.64, 1.55)	1.05 (0.52, 2.16)	0.96 (0.40, 2.27)	0.42 (0.15, 1.19)	0.92 (0.36, 2.29)	1.16 (0.55, 2.32)	1.13 (0.59, 2.18)	1.11 (0.61, 2.01)
1.00 (0.64, 1.55)	**cDMARDs**	1.05 (0.60, 1.86)	0.95 (0.45, 2.01)	0.42 (0.16, 1.07)	0.91 (0.41, 2.05)	1.16 (0.63, 1.99)	1.12 (0.71, 1.80)	1.11 (0.73, 1.65)
0.95 (0.46, 1.93)	0.95 (0.54, 1.67)	**CZP+MTX**	0.90 (0.35, 2.27)	0.40 (0.13, 1.20)	0.87 (0.33, 2.32)	1.09 (0.48, 2.36)	1.06 (0.52, 2.23)	1.04 (0.52, 2.10)
1.04 (0.44, 2.48)	1.05 (0.50, 2.20)	1.11 (0.44, 2.83)	**ETN+MTX**	0.44 (0.13, 1.43)	0.96 (0.32, 2.83)	1.23 (0.46, 3.03)	1.19 (0.50, 2.86)	1.16 (0.49, 2.69)
2.36 (0.84, 6.82)	2.36 (0.93, 6.17)	2.51 (0.84, 7.61)	2.25 (0.70, 7.61)	**GOL**	2.16 (0.90, 5.64)	2.75 (0.92, 8.25)	2.66 (0.96, 7.61)	2.61 (0.94, 7.39)
1.08 (0.44, 2.75)	1.09 (0.49, 2.46)	1.15 (0.43, 3.06)	1.04 (0.35, 3.10)	0.46 (0.18, 1.12)	**GOL+MTX**	1.27 (0.46, 3.35)	1.21 (0.48, 3.16)	1.20 (0.49, 2.97)
0.86 (0.43, 1.80)	0.86 (0.50, 1.58)	0.91 (0.42, 2.10)	0.81 (0.33, 2.18)	0.36 (0.12, 1.08)	0.79 (0.30, 2.16)	**IFX+MTX**	0.96 (0.48, 2.12)	0.95 (0.49, 1.95)
0.89 (0.46, 1.68)	0.90 (0.55, 1.40)	0.94 (0.45, 1.93)	0.84 (0.35, 1.99)	0.38 (0.13, 1.04)	0.83 (0.32, 2.08)	1.04 (0.47, 2.08)	**TCZ**	0.98 (0.61, 1.52)
0.90 (0.50, 1.65)	0.90 (0.61, 1.36)	0.96 (0.48, 1.93)	0.86 (0.37, 2.03)	0.38 (0.14, 1.06)	0.84 (0.34, 2.03)	1.05 (0.51, 2.05)	1.02 (0.66, 1.63)	**TCZ+MTX**

*cDMARDs, conventional disease-modifying antirheumatic drugs; MTX, methotrexate; ADA, adalimumab; CZP, certolizumab; ETN, etanercept; GOL, golimumab; IFX, infliximab; TCZ, tocilizumab; PBO, placebo*.

### Ranking with SUCRA value

Table 4 showed the results of ranking probabilities in terms of each outcome. As for the efficacy outcomes, TCZ+MTX ranked first on ACR50, ACR70 and ranked second on ACR20 and remission, which indicated its best performance among all treatments. With respect to alternative treatment options, ETN+MTX and IFX+MTX also performed well due to their higher SUCRA values compared with other treatments. And PBO ranked last on all efficacy outcomes as expected. Regarding the safety outcomes, though ETN ranked first followed by GOL for AEs and IFX+MTX, TCZ+MTX had the similar highest values for SAEs according to the SUCRA ranking, the conclusion about the relative safety of each concerning treatment seemed not so credible if we combined the results of forest plots. What's more, due to the lack of data, some SUCRA results were missing which may also reduce the reliability of the outcomes.

**Table 4:** The SUCRA value of different treatments on each outcome.

	**ACR20**	**ACR50**	**ACR70**	**Remission**	**AE**	**SAE**
ADA	0.144	0.157	0.244	0.080		
ADA_MTX	0.603	0.576	0.624	0.347	0.449	0.449
cDMARDs	0.290	0.274	0.277	0.130	0.613	0.437
CZP_MTX	0.494	0.480	0.523	0.773	0.312	0.509
ETN	0.198	0.146	0.186		0.905	
ETN_MTX	0.806	0.794	0.757	0.530	0.591	0.418
GOL	0.351	0.355	0.227	0.299	0.742	0.039
GOL_MTX	0.564	0.501	0.482	0.489	0.324	0.405
IFX_MTX	0.717	0.721	0.714	0.568	0.373	0.603
PBO	0.004	0.001	0.000		0.473	
TCZ	0.607	0.653	0.665	0.622	0.532	0.578
TCZ_MTX	0.723	0.841	0.803	0.739	0.186	0.562

*cDMARDs, conventional disease-modifying antirheumatic drugs; MTX, methotrexate; ADA, adalimumab; CZP, certolizumab; ETN, etanercept; GOL, golimumab; IFX, infliximab; TCZ, tocilizumab; PBO, placebo*.

### Consistency analysis

Figures S1–S6 showed the results of node-splitting analysis and their corresponding heat plots. All the *P*-value was larger than 0.05 which revealed that there was no statistical inconsistency between direct and indirect comparisons among all outcomes. The same results came from the heat plots, which also contributed to the reliability of this NMA.

## Discussion

RA was a type of chronic inflammatory arthritis, which would have negative effects on patients' living quality. Moreover it can lead to functional limitations and employment obstacle (Singh et al., 2009). Many kinds of medications have been introduced to cure RA, including conventional DMARDs (like MTX), biologics (like IFX, ETN, ADA, and PCZ), and other concerning medicines (Jansen et al., 2014). The mechanism of biologic agents is that they can target TNF-α, IL-1, IL-6, T cells, or B cells, and significantly inhibit the damage of joint. In our NMA, we collected data from 20 eligible trials of 9,047 patients with RA who were cDRAMDs-naïve. Eleven interventions along with PBO were compared simultaneously on both efficacy and safety.

Four outcomes concerning efficacy were measured, including ACR20, ACR50, ACR70, and remission. As was shown in this NMA, all of the 11 therapies worked notably better than PBO. Moreover, we can also find that the combination of biological agents with MTX might be superior to monotherapy of cDMARDs, particularly TCZ+MTX, which performed well in all four outcomes with respect to efficacy. In addition, the monotherapy of TCZ also ranked roundly well in all outcomes despite that it was inferior to combination of TCZ and MTX. The results above were consistent to former trials. For instance, a double-blind, 2-year study (Kremer et al., 2011) containing 1196 RA patients indicated that TCZ+MTX had better efficacy on helping patients slow down the joint damage and improving their body function than MTX alone. IL-6 is a pleiotropic cytokine that can regulate the immune response, hematopoiesis, inflammation, and bone metabolism through combining with IL-6 receptor. The constitutive overproduction of IL-6 is considered to play a pathological role in RA (Nishimoto et al., 2007). Correspondingly, TCZ is a humanized monoclonal antibody that can suppress the bindling of IL-6 to membrane expressed IL-6 soluble receptors, thus preventing the pro-inflammatory activities of IL-6 or IL-6 receptor signaling (Kremer et al., 2011), which might explain TCZ as an effective medication for RA.

Apart from that, ETN+MTX and IFX+MTX also performed pretty well as alternative treatment options. According to previous studies, TNF is a kind of soluble protein playing an important role in RA. It is believed that TNF can lead to continuous occurrence of inflammatory response and progressive destruction of cartilage and bone (Brennan et al., 1992). Both ETN and IFX are TNF antagonists, which can be specifically combined with TNF-α and then break the activity of it to achieve the goal of controlling inflammation and continuing to alleviate the symptom of RA (Emery et al., 2008a; Lee and Bae, 2016).

What more, when comparing the combinations of cDMARDs and biological agents with monotherapy of biologic agents, according to the results, the combination therapies, showed the much higher efficacy than the corresponding biologic medicine alone. While as for the safety outcomes, the results didn't show the significant difference, which also indicated the superiority of combination therapies.

When choosing an appropriate treatment, not only the efficacy, but also the safety ought to be considered. When it came to safety, the results of AEs together with SAEs were measured in this NMA. Though ETN ranked first with respect to avoiding adverse events and those medications which performed well in the outcomes of efficacy did not rank well in safety according to the SUCRA ranking, the result could not be so responsible due to the outcomes of forest plots which indicated that there is no statistical difference between those combinations of drugs and cDRAMDs alone.

Moreover, the main adverse events of those therapies included rhinopharyngitis, respiratory tract disorder, pathology of skin and soft tissue, gastrointestinal side effects and so on (Campbell et al., 2011). A study conducted among 48,676 participants (Singh et al., 2011) concluded that biologics were connected with significantly higher rates of tuberculosis reactivation, serious infections, total AEs and withdrawals resulted by AEs, which is worthy noticing. Therefore, due to the potential adverse effects, the use of effective interventions still needs further evaluation.

However, there are inevitably some limitations in our research. Firstly, the sample size of some outcomes was relatively small and duration time was relatively short, thus we could not obtain sufficient evidence to get general results. Secondly, most of the RCTs included in our NMA compared medications with cDMARDs, as a consequence, some direct comparisons between other treatments could not been achieved and we could not get a further understanding in the inconsistency among these RCTs. For instance, we were not able to compare ETN+MTX and TCZ+MTX directly. Besides, there were some confounding factors while analyzing the outcomes, which may have some effects on study. For example, doses across treatments had not been accounted for in this analysis. In addition, the different stages of RA might also affect the result of study since patients in different stages appeared varied symptoms and responded differently to medications.

In summary, based on the studies we selected, our NMA provided a systematic evaluation on the efficacy and safety of therapies on rheumatoid arthritis. Our NMA concluded that TCZ+MTX was potentially the most preferable treatment for RA, with ETN+MTX and IFX+MTX as alternative treatment options. However, considering the adverse effects, it should be introduced with caution and more advanced studies should be carried out to find out the most appropriate way for treating RA.

## Author contributions

WC, YG, and HC: Substantial contribution to the conception and design of the work; YC: Analysis and interpretation of the data; WC, YG, XW, and YY: Drafting the manuscript; MW: Revising the work critically for important intellectual content; Final approval of the work: all authors.

### Conflict of interest statement

The authors declare that the research was conducted in the absence of any commercial or financial relationships that could be construed as a potential conflict of interest.

## Supplementary material

Figure S1 The results of consistency analysis by note-splitting plot and heat plot of ACR20.

Figure S2 The results of consistency analysis by note-splitting plot and heat plot of ACR50.

Figure S3 The results of consistency analysis by note-splitting plot and heat plot of ACR70.

Figure S4 The results of consistency analysis by note-splitting plot and heat plot of remission.

Figure S5 The results of consistency analysis by note-splitting plot and heat plot of adverse events.

Figure S6 The results of consistency analysis by note-splitting plot and heat plot of serious adverse events.
