# Supplementary figures and images for: The Efficacy and Safety of Mainstream Medications for Patients With cDMARD-Naïve Rheumatoid Arthritis: A Network Meta-Analysis
Source: Front Pharmacol. 2018 Mar 21;9:138. doi: 10.3389/fphar.2018.00138 (PMC5871709; doi:10.3389/fphar.2018.00138)

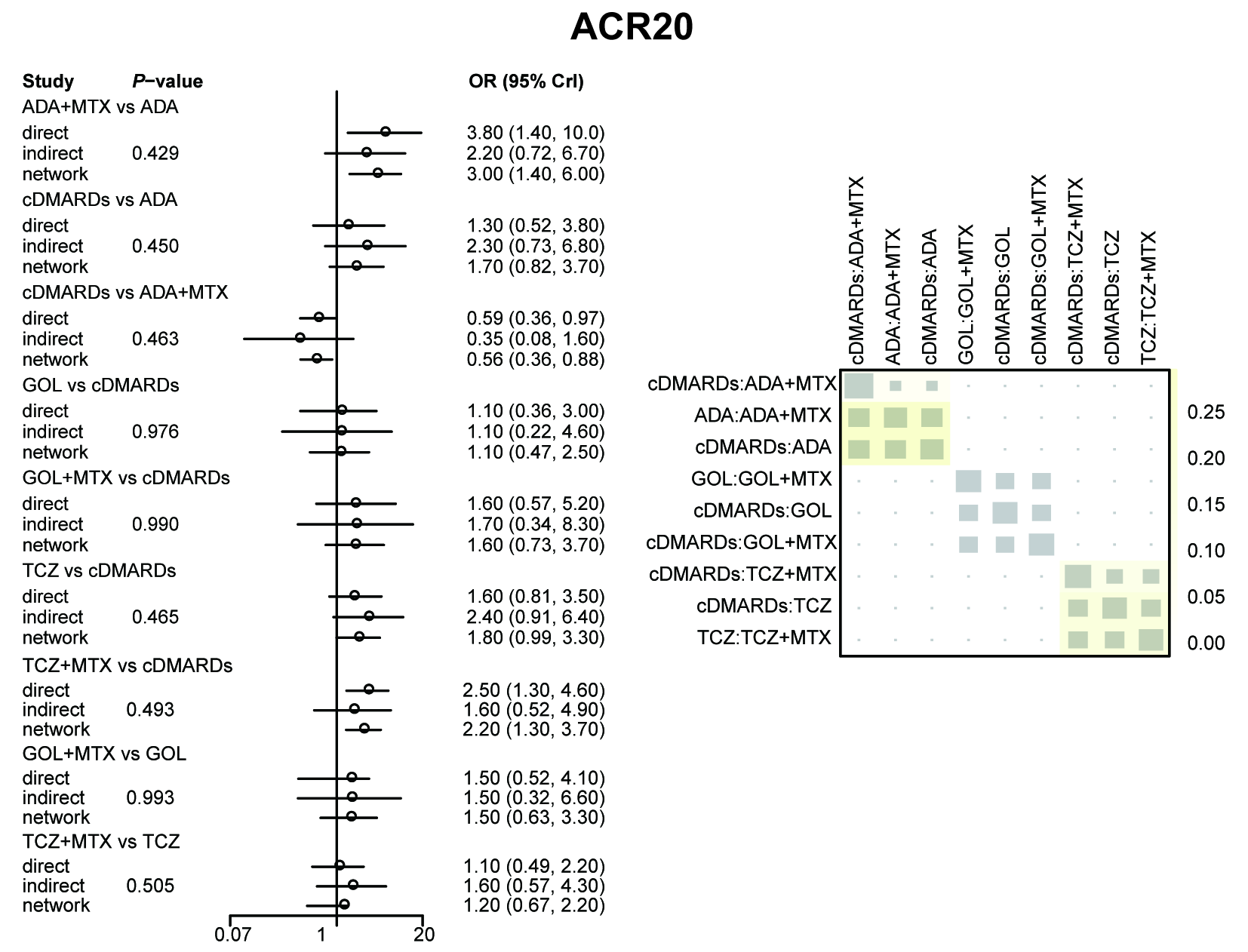

Supplement: Figure S1 — The results of consistency analysis by note-splitting plot and heat plot of ACR20. [file Image1.TIF]

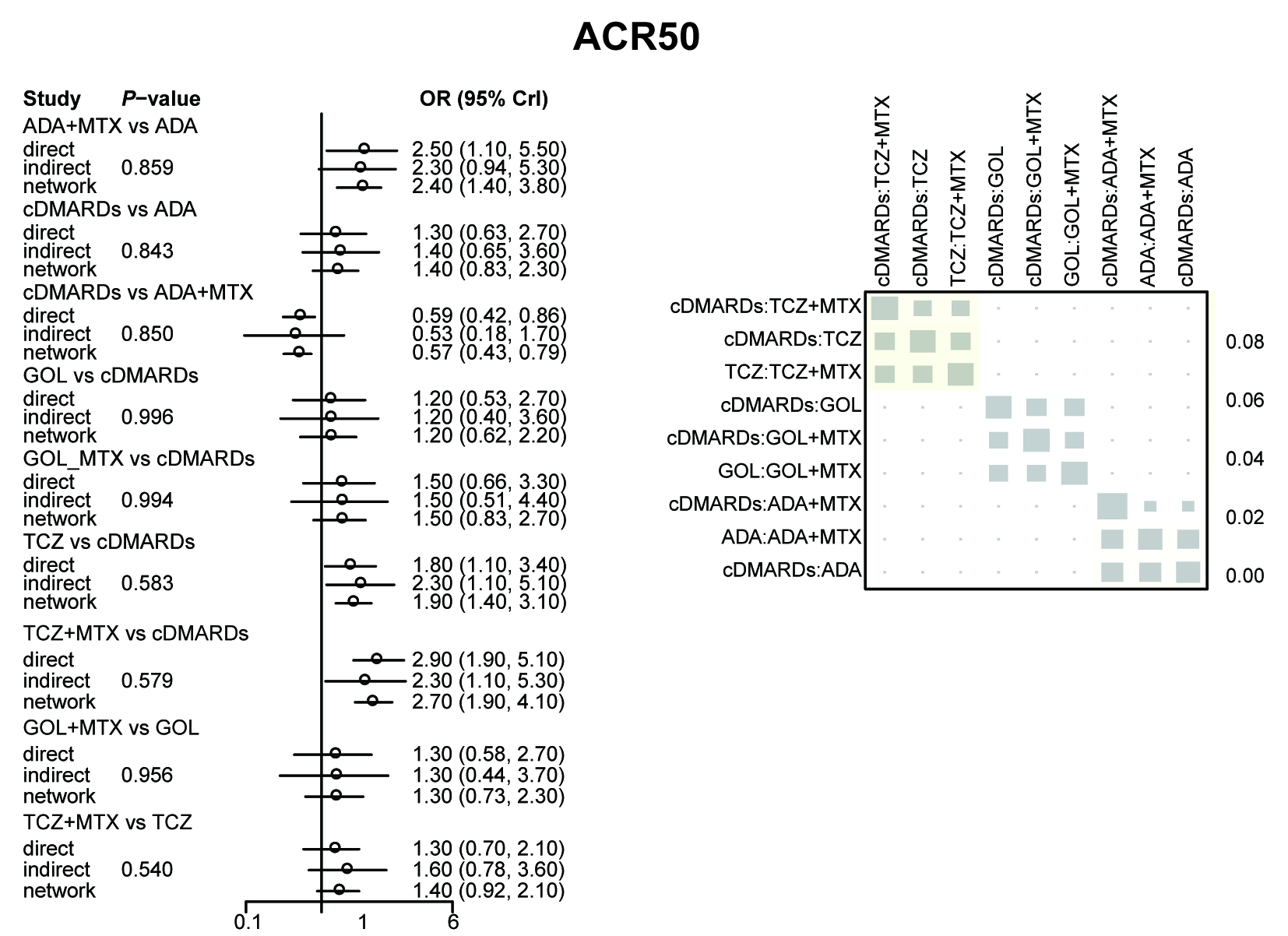

Supplement: Figure S2 — The results of consistency analysis by note-splitting plot and heat plot of ACR50. [file Image2.TIF]

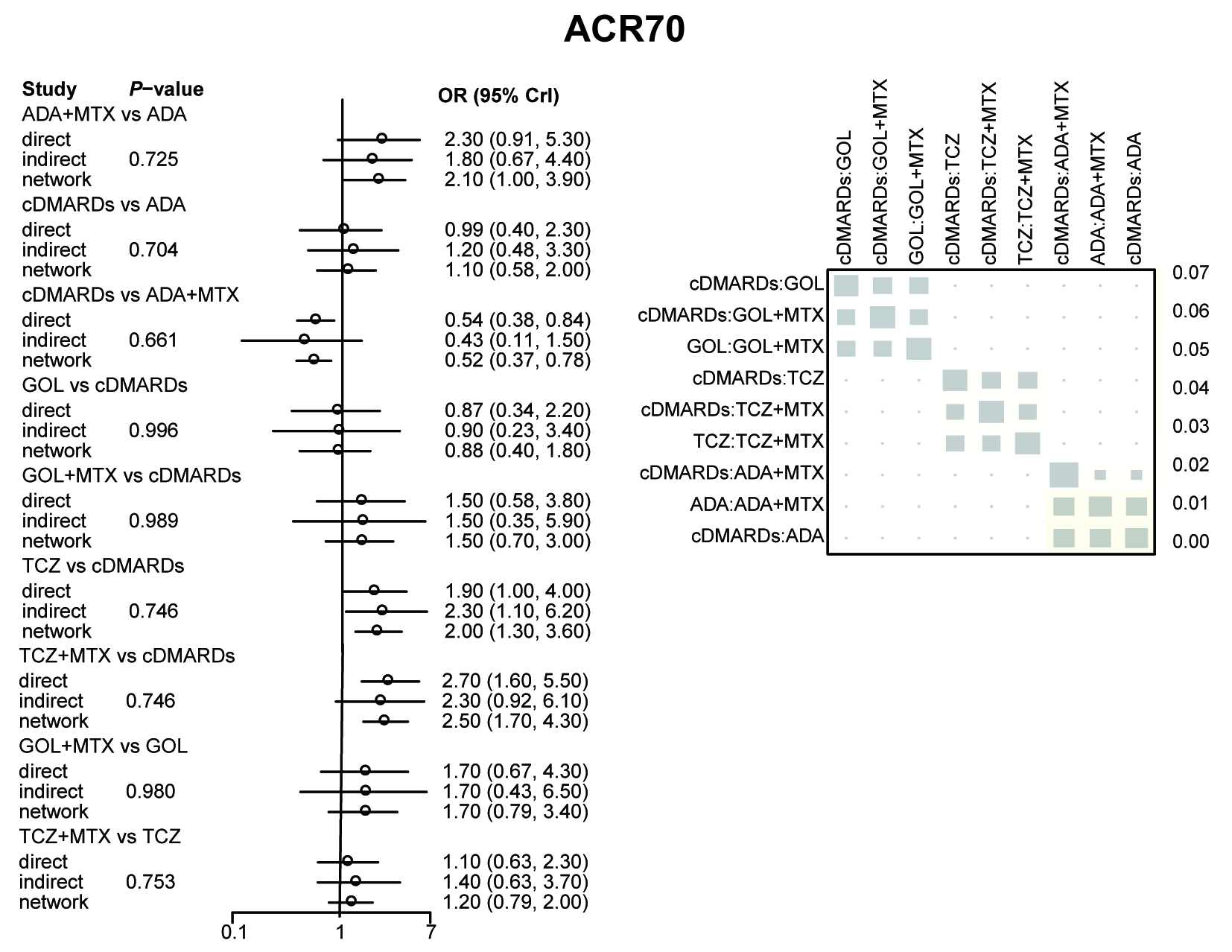

Supplement: Figure S3 — The results of consistency analysis by note-splitting plot and heat plot of ACR70. [file Image3.TIF]

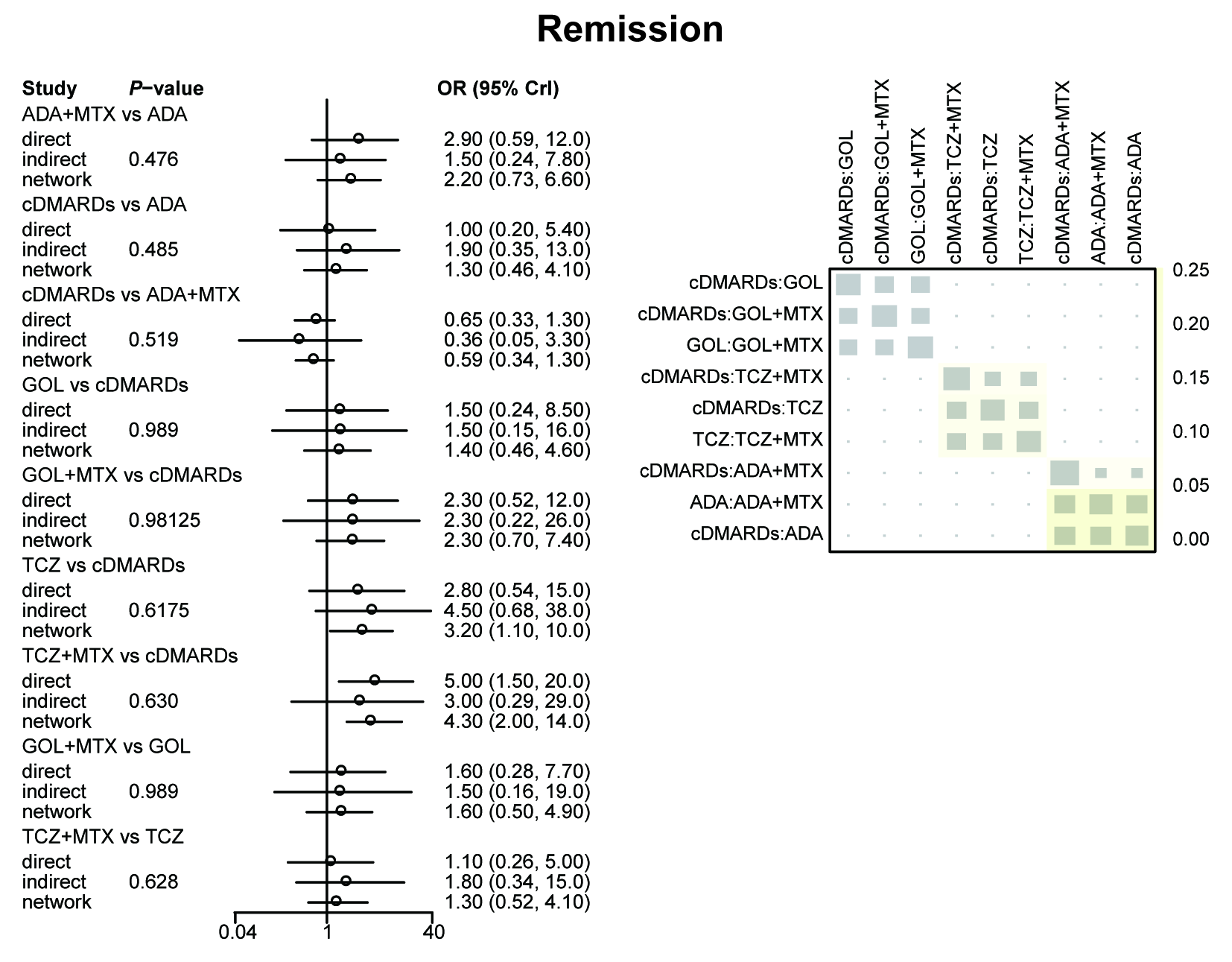

Supplement: Figure S4 — The results of consistency analysis by note-splitting plot and heat plot of remission. [file Image4.TIF]

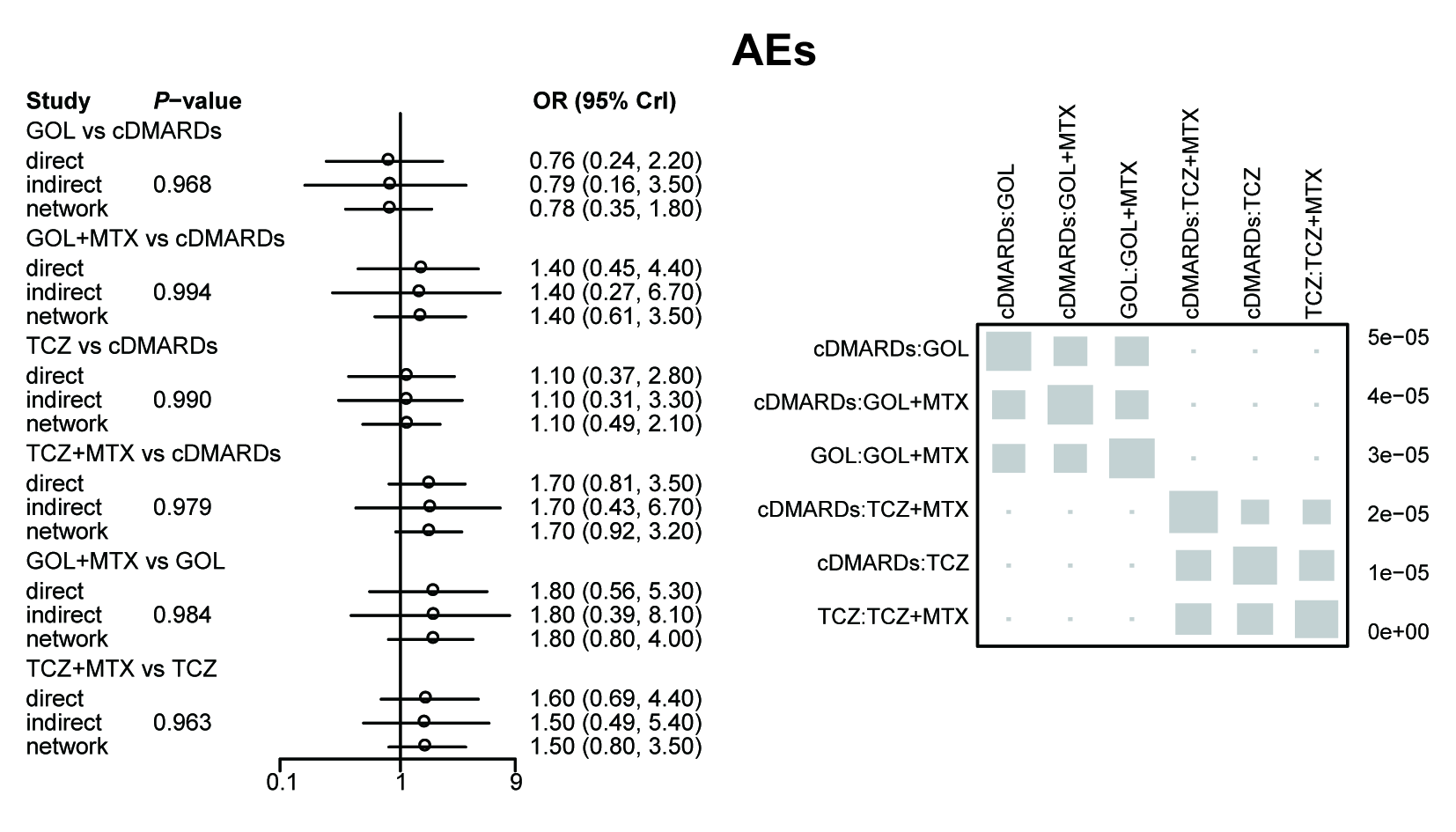

Supplement: Figure S5 — The results of consistency analysis by note-splitting plot and heat plot of adverse events. [file Image5.TIF]

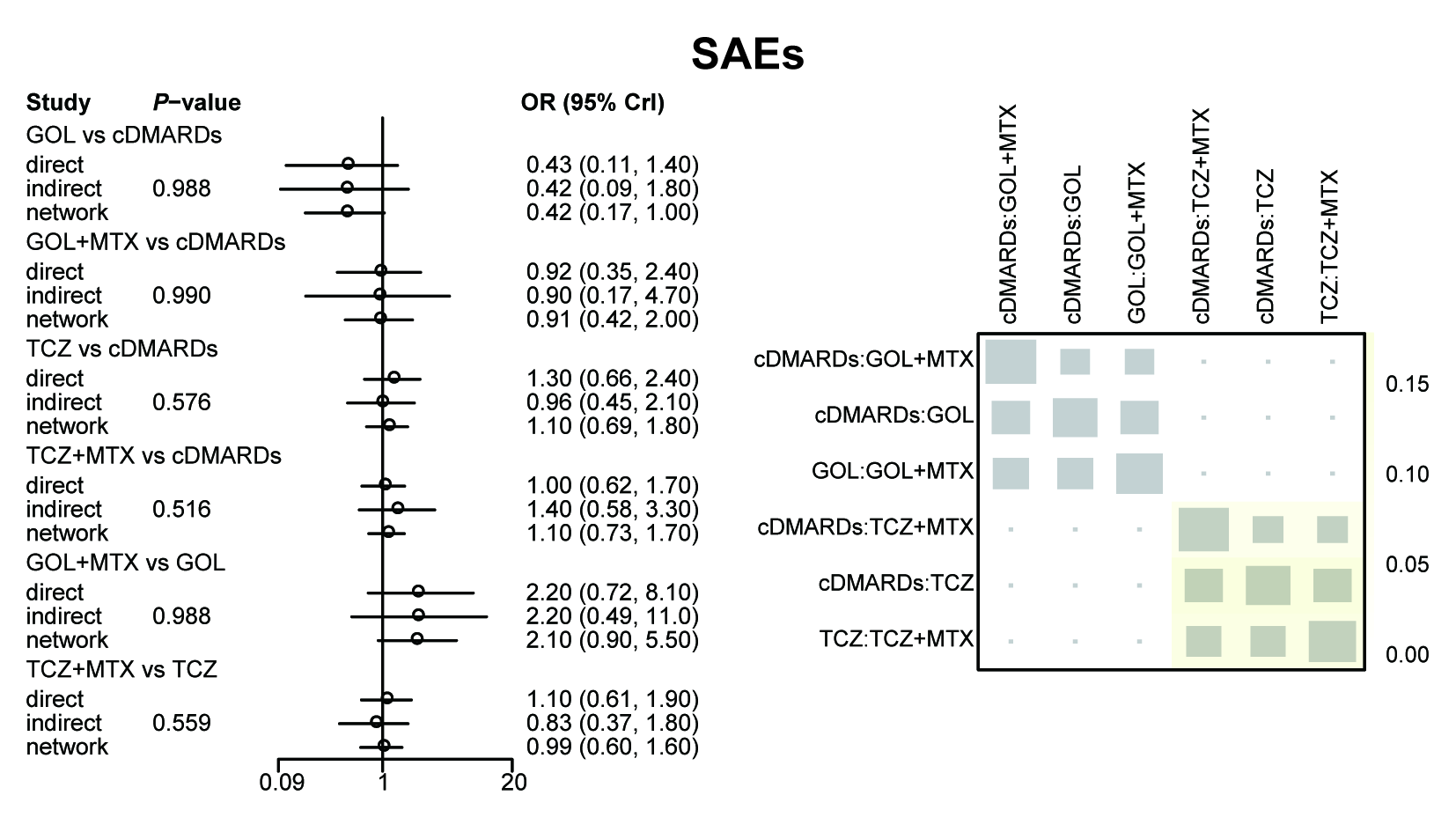

Supplement: Figure S6 — The results of consistency analysis by note-splitting plot and heat plot of serious adverse events. [file Image6.TIF]
